# Effectiveness of spiritual care training to enhance spiritual health and spiritual care competency among oncology nurses

**DOI:** 10.1186/s12904-019-0489-3

**Published:** 2019-11-26

**Authors:** Yanli Hu, Miaorui Jiao, Fan Li

**Affiliations:** 1 School of Nursing, Jilin University, Changchun, China; 2 Department of Digestive Radiotherapy, Affiliated Tumor Hospital of Zhengzhou University Henan Cancer Hospital, Zhengzhou, China; 3 Department of Pathogenobiology, The Key Laboratory of Zoonosis Research, Chinese Ministry of Education, College of Basic Medicine, Jilin University, Changchun, China; 4 The Key Laboratory for Bionics Engineering, Ministry of Education, Jilin University, Changchun, Jilin China; 5 State Key Laboratory of Pathogenesis, Prevention and Treatment of High Incidence Diseases in Central Asia, Xinjiang, China; 6 College of Basic Medical Science, Jilin University, No. 126 Xinmin Street, Changchun, 130021 China

**Keywords:** Nurses, Spiritual care, Spiritual health, Spiritual care competency

## Abstract

**Background:**

Although spiritual care is a basic element of holistic nursing, nurses’ spiritual care knowledge and abilities are often unable to satisfy patients’ spiritual care needs. Therefore, nurses are in urgent need of relevant training to enhance their abilities to provide patients with spiritual care.

**Design:**

A nonrandomized controlled trial.

**Objective:**

To establish a spiritual care training protocol and verify its effectiveness.

**Methods:**

This study recruited 92 nurses at a cancer treatment hospital in a single province via voluntary sign-up. The nurses were divided into two groups—the study group (45 people) and the control (wait-listed) group (47 people)—using a coin-toss method. The study group received one spiritual care group training session every six months based on their routine nursing education; this training chiefly consisted of lectures by experts, group interventions, clinical practice, and case sharing. The control group participated in monthly nursing education sessions organized by the hospital for 12 continuous months.

**Results:**

After 12 months of intervention, the nurses in the study group had significantly higher overall spiritual health and spiritual care competency scores as well as significantly higher scores on all individual dimensions compared with those in the control group (*P* < 0.01).

**Conclusions:**

A spiritual care training protocol for nurses based on the concept of mutual growth with patients enhances nurses’ spiritual well-being and spiritual care competencies.

## Background

Although the debate on ‘defining’ spirituality [1, 2] is long-standing, spiritual health (also known as spiritual well-being: in the Chinese cultural context, the construct of spiritual well-being is the same as spiritual health [3, 4], and this is also consistent with the general view that “spiritual health is considered as one of the important dimensions of wellness [5]“) in the present study refers to “the state of an individual’s affirmation of the meaning of his or her own life; understanding and affirmation of the value of oneself, others, and the environment; the ability to connect harmoniously with others and the environment; the possession of inner resources and strength; and ability to adapt to adversity.” [1–6]. This definition represents the existential meaning of spiritual health. Spiritual health often includes six aspects [7–9]: an individual’s relationship with him or herself, with others, and with the environment; his or her beliefs; the ability to overcome adversity; and the meaning of life. As one of the core elements of quality of life, spiritual health is of great importance [10, 11]. Research has shown that the enhancement of nurses’ spiritual health not only boosts their personal satisfaction with life [12, 13] but also reduces job burnout [14] and assists them with providing spiritual care to patients in their clinical work. Nurses are the chief providers of spiritual care to patients [15–17], and a close relationship exists between nurses’ attitude and ability to provide spiritual care to patients and their perceptions of spirituality and spiritual health [18–21].

Spiritual care encompasses an attitude and behaviour shaped by nurses’ spiritual nursing values [22, 23] (especially the affirmation of attributes such as human dignity, goodness, benevolence, peace of mind, warmth, self-care, and care for others) and consists of care that reflects individuals’ cultures and beliefs provided after assessing their spiritual needs and challenges [24]. Spiritual care also consists of nursing methods or activities that rely on the provision of company or care, listening, or religious activities that correspond to patients’ beliefs to help them to achieve better physical, mental, social, and spiritual health and comfort [17, 25] The purpose of spiritual care is to ease patients’ difficulties at the spiritual level and help them find the meaning of life, self-actualization, hope, creativity, faith, trust, peace, comfort, prayer, and the ability to love and forgive in the midst of suffering and disease [17, 26]. Additionally, spiritual care seeks to help patients to face their fears of death, mitigate the uncertainty and discomfort of the treatment process, and regain their inner peace [27–29]. In clinical work, spiritual care education and training helps nurses to understand patients’ senses of honour, values, and experience to express kind concern for their patients, ease patients’ stress and tension, provide them with spiritual well-being and serenity [30] and let them find meaning and purpose amidst adversity [17, 31]. Under this care, patients can explore strategies to overcome their illnesses as well as strengthen their physical, social, and psychological health, thereby improving their quality of life and state of health [17, 23, 31, 32]. Spiritual care is a core element of holistic nursing and has already been incorporated into nursing education and practice [33–36]. In addition, the ability to provide spiritual care to patients is increasingly considered a major occupational skill for nurses [19, 23].

In clinical work, however, nurses have insufficient knowledge and understanding of spiritual care and inadequate spiritual care competencies, which has led to patients’ spiritual needs often remaining unmet [37–39]. Research has shown that medical personnel who have undergone spiritual care training are more likely to meet patients’ spiritual needs when providing spiritual care [39–44]. Currently, research on nurses’ spiritual health and spiritual care competencies remains at a preliminary stage [34, 45], and little relevant interventional research has been conducted. As a consequence, scientifically based and effective intervention protocols must be drafted to boost nurses’ levels of spiritual care. The aim of this study was to evaluate the effects of a spiritual care programme for oncology nurses. We investigated whether nurses gain benefits from this training such as positive changes in their spiritual health and higher levels of spiritual care competencies.

## Methods

This was a nonrandomized study, and nurses were recruited by voluntary participation. Participants were incompletely randomly assigned to an intervention group or a control group.

### Participants

From September 2017 to October 2018, 92 clinical nurses at a cancer treatment hospital in a single province were selected based on a survey of existing conditions. These nurses were assigned to a study or a control group via a coin-toss method. The following inclusion criteria were considered for the current study: registered nurses who had been engaged in clinical work for ≥5 years (The present study is part of a series of research projects. The majority of nurses with more than five working years are mostly clinical teachers in China, which will facilitate the training of other nurses and nursing students in the later period and can create an environment to foster the provision of spiritual care.), signed an informed written consent form and voluntarily participated in the study. The exclusion criteria were as follows: nurses receiving other advanced training; individuals who might be expected to drop out of the study due to pregnancy, retirement, or transfers; and individuals joining part way through the intervention. No significant differences were found with regard to age, work seniority, occupation, religious beliefs, etc. between the two groups. The baseline data for the nurses were uniform across the two groups (see Additional file 1: Table S1).

### Procedure

#### Establishment of an intervention team

The members of the intervention team consisted of two palliative nursing education experts and three clinical head nurses who specialize in spiritual care. In addition, one statistical expert with statistical analysis skills was recruited who participated in the analysis of the data but not in the intervention process.

#### Drafting of the intervention protocol

The intervention methods included lectures and instruction, case sharing, group discussion, and individual psychological counselling. Yao Jianan et al. [46] suggest that spiritual care education should include life-and-death education as well as hospice care education. Some scholars [47] have incorporated religious and communication skills into teaching. Timmins et al. [48] concluded that spiritual content education includes personal understanding of spirituality and spiritual concepts and identification of patients’ spiritual needs. Ross et al. [23] showed that nurses’ personal beliefs and values and their spirituality and sensitivity levels can affect spiritual care outcomes. Baldacchino [49] proposed self-reflection, case studies, and small-group discussions to promote nurses’ spirituality and the understanding of spiritual care to improve their competencies’ in spiritual care.

The spiritual care training protocol was drafted based on a literature review, expert recommendations, and the results of a current status survey. This study’s preliminary examination of the literature [18, 43, 45, 50] with clinical nurses revealed that nurses have great need for spiritual care training, which was reflected in the fact that the nurses realized that their spiritual health and self-perceptions of spirituality must be improved and that the long-term clinical care of patients with cancer had made the nurses aware that although these patients tend to have high spiritual care needs, the nurses’ own spiritual care competencies and experience were insufficient, which was (importantly) due to inadequate training. Using the nurses’ spiritual health and growth as a point of entry, we designed a spiritual care training protocol that focused on nurses’ spiritual health and spiritual care competencies. This protocol used strategies such as “spiritual care education fosters spiritual practice” [51], “in order to convey the mystery of life to others, people need to perceive the paradox within themselves” [52], and “spiritual growth occurs among people participating in service-learning” [53] to guide the nurses to view themselves more clearly, explore the mysteries of life, and learn about the operating rules of the spiritual world in detail. In addition to providing the nurses with positive professional knowledge and attitude, the protocol sought to encourage the nurses to uncover their full potential through a “focus on the establishment of a positive mood and sense of happiness, an emphasis on holistic development, and the goal of uncovering, cultivating, and realizing individuals’ strengths and potential” [54] while boosting their spiritual health and promoting better spiritual care skills.

This study design included a 12-month spiritual care training protocol for nurses based on the hospital’s general nursing education; this protocol, which was implemented from September 14, 2017, to October 15, 2018, consisted of spiritual care training classes for two weeks every six months, two case sharing sessions each month, and one personal growth book discussion session each month. The spiritual health, spiritual care competencies, emotional regulation, and level of psychological resilience of the nurses in the two groups were tested before and after training. The members of the control group participated in one intensive nursing service training session each month during the training period.

(1) Study group protocol. The intervention group participated in the one session/month concentrated study organized by the hospital in the same period as the control group. In addition, all members of the study group concentrated on the training for two sessions, once every six months, five days/session, eight hours/day. The lecturers were senior teaching supervisors of clinical spiritual care education and pastoral counselling in China. After the centralized training, organized activities occurred twice a month, including one case sharing, three hours; personal growth reading activities, one session, two hours each session, including two books in the training period: “You Can Heal Your Life”(Louise Hay, US) and “Being Mortal: Medicine and What Matters in the End”(Atul Gawande, US). The time and place of the event were fixed. The “spiritual care training classes” of this training were incorporated into the workday and approved by the hospital nursing manager for continuing education. The team members participating in the training attended two weeks of special spiritual care group study every year. The time of “case sharing sessions” was the time used for the monthly seminar. The “the personal growth book discussion” time was after dinner every Wednesday, and this session was the only one of the training programme that was outside the workday. Each activity was led by a spiritual care training team leader responsible for organizing and implementation. The team leader had three deputy senior titles and a clinical head nurse who had obtained the qualification of a national second-level counsellor. This protocol included life-and-death education, suicide prevention strategies, end-of-life care, spiritual growth, spiritual care cognition and practice, etc. (see Additional file 1: Table S2).

(2) Control group protocol. The control group only participated in the centralized study of the hospital for one session/month during this period. Each session lasted two hours. The training received by the control group included nursing research training and the care of commonly observed psychological problems among patients with cancer. To ensure equal treatment, if the effectiveness of this study’s spiritual care training protocol was verified after the 12th month, then the intervention would be continued to provide the control group nurses and the other nurses in the hospital with the same specialized spiritual care training.

#### The content of the intervention

The content of the spiritual care education curriculum included a group pledge and the sharing of feelings, empathy training, positive spiritual education fostering personal growth, reflective logs, the law of attraction (the law of attraction means that when thoughts are concentrated in a certain field, people and things related to this field will be attracted to a person with certain qualities [55]; this conception helps one to find stability and security in a changing and challenging world [56] and shows how one person can use spiritual tools to change one’s mindset from a fearful one into a more confident positive approach to the world [57, 58]), suicide prevention, end-of-life care, and life-and-death education.

### Implementation of the intervention protocol

#### Assessment period

Prior to the start of the intervention, the members of the intervention team performed a baseline survey of all of the participating nurses. The intervention protocol arrangements and the amounts of time required were explained to the nurses before the intervention, and their informed consent was obtained. The nurses’ baseline data were then analysed. Authorization of use of all questionnaires was obtained from the authors of the original questionnaires.

#### Intervention period

The nurses received spiritual care training chiefly in accordance with the intervention protocol developed for this study during the intervention period. During the two-week intensive training periods that focused on three topics each period, the intervention format chiefly consisted of experts’ lectures and interactive classroom discussion. Prior to the conclusion of the intensive intervention, the trainees were asked to receive a one-month reinforcement training session and were informed about enhanced training assignments and completion requirements. After each intervention period concluded, the nurses completed a training class feedback questionnaire to assess their training results.

#### Evaluation period

After the spiritual care training concluded, the Spiritual Health Scale (SHS) and the Chinese version of the Spiritual Care Competency Scale (C-SCCS) were administered to both groups, and one-on-one interviews were conducted to assess the degree to which the goals of the intervention protocol had been realized.

### Assessment instruments

#### General background survey form

The general background survey form included items regarding age, work seniority, level of education, title, monthly income, technical titles, position, religion, and assessment of one’s own work.

#### Spiritual health scale

The SHS is a spiritual well-being scale developed by the Taiwanese scholars Ya-chu Hsiao et al. [59] based on a sample consisting of nurses. This scale comprises 24 questions across five dimensions: bonding with others, searching for meaning, overcoming adversity, religious faith, and self-knowledge. This scale has a Cronbach α coefficient of 0.93, with subscale values ranging from 0.77 to 0.89 [60]. The scale’s use has reached a level of maturity, and it has been steadily optimized.

#### Spiritual care competency scale

Leeuwen et al. drafted this scale [47]; it contains 27 items across six dimensions, is scored using a five-point Likert scale, and has excellent validity and reliability [47]. The Chinese version was translated and culturally localized by the study team with the permission of Dr. Leeuwen. It was evaluated using the Exploratory factor analysis (EFA) and Confirmatory factor analysis (CFA) method [34] and was divided into three dimensions using factor analysis based on the conceptual framework of the original scale: assessment, implementation, professionalization, and quality improvement of spiritual care (AIPI); personal and team support (PTS); and attitudes towards patient spirituality and communication (ATPSC). The Cronbach α coefficients of these dimensions were 0.93, 0.92, and 0.89, respectively [34].

### Statistical methods

*IBM SPSS* 23.0 was used for data analysis. We employed independent samples testing to compare the two groups’ preintervention test scores. The paired-samples *t*-test was used to compare the preintervention and postintervention scores of the two groups, and the independent samples *t*-test was used to compare the postintervention scores of the two groups; *P* < 0.05 was considered significant, and the eta squared (*η*^2^) was used to evaluate the effect sizes of the intervention.

## Results

### Sample characteristics

Additional file 1: Table S1 shows the comparison of demographic characteristics between nurses in the two groups. A total of 92 nurses completed the experiment. All returned questionnaires were suitable for this study. All nurses were female. The average length of employment in the study group was 18 years, and that in the control group was 16 years. The basic characteristics of the participants are summarized in Additional file 1: Table S1.

#### Between-group comparisons of the spiritual health and spiritual care competency before and after intervention

The results indicated that the study group had higher total spiritual health and spiritual care competency scores as well as higher scores for their individual dimensions than the control group (*P* < 0.05). However, the score of the “Self-understanding” dimension of the spiritual health scale was not higher in the study group than in the control group (*P* > 0.05). The effect sizes of sub-scales of the two scales after intervention ranged from 0.04 to 0.28. See Additional file 2: Table S3.

#### Comparison between the preintervention and postintervention spiritual health and spiritual care competency scores of the nurses in the study group and control group

The results indicated that the overall spiritual health and spiritual care competency scores and their subscale scores in the study group were higher after the intervention (*P* < 0.05), and the effect values of the study group before and after the intervention ranged from 0.23 to 0.88 (see Additional file 2: Table S4).

## Discussion

### Spiritual care training enhances nurses’ spiritual health and spiritual care competencies

The above study results indicate that the nurses in the study group had significantly higher total spiritual health and spiritual care competency scores as well as significantly higher individual dimension scores after the intervention with a moderate to intense effect. In addition, compared with the control group, the study group showed significantly better spiritual health and spiritual care competency scores as well as significantly better individual dimension scores following the intervention. Although the control group had higher spiritual care competency scores prior to the intervention (this result is likely because the nurses in the control group were slightly younger than those in the study group and therefore had less exposure to the spiritual care concept), the nurses in the study group displayed greater spiritual care competency than the nurses in the control group after the 12-month intervention. Based on the results of a survey of the prevailing state of affairs and using spiritual growth as a point of entry, this study designed a spiritual care training protocol for clinical nurses to address their perceptions of spirituality and spiritual care competencies. The results of this study indicated that this protocol supported specific achievements.

The goal of spiritual care training for nurses is to help them understand the methods and techniques that they can use to provide spiritual care to their patients. During this process, nurses’ self-perceptions of spirituality are inevitably raised, and true spiritual clinical nursing practice will have a major, albeit intangible, effect on nurses’ own spiritual health and their cognition of additional psychological aspects [41, 43, 49, 60]. Through the group intervention, the nurses expressed their feelings, which gave them an opportunity to review their own spiritual needs. When nurses transform the knowledge and skills that they learn in training into active, conscious clinical practice (e.g., life-and-death education and the end-of-life care process of patients with terminal cancer), the nurses’ own perspectives of life and death as well as their attitudes towards the things around them will also change.

Furthermore, the group pledge and sharing of feelings provide an appropriate setting and environment for the sharing and expression of the nurses’ spiritual experiences. After being in contact with each other for a certain period of time, the team members formed a cooperative learning team that enjoyed mutual growth. This study’s effort to employ a “spiritual education fostering personal growth” to increase the nurses’ spiritual awakening and use of personal growth methods to inspire awareness of and search for positive life goals as well as realize the concept of a spiritually and behaviourally healthy person significantly enhanced the nurses’ level of spiritual well-being, including such aspects as living a meaningful life and understanding oneself.

### Clinical relevance

Motivated by the growing importance placed on the provision of spiritual care to patients, research on spiritual care has recently increased [34]. However, although previous research has shown that both patients and their family members have great spiritual needs and that medical personnel must show concern for and satisfy these needs, this issue has not received sufficient attention in nursing practice [37, 61]. One obstacle to spiritual care practice is that nurses—the chief healthcare providers—are insufficiently prepared to take on this role because of their inadequate education in this area [28–31, 43, 45]. To provide more effective spiritual care to patients, it is urgent that nurses receive education or training to improve their spiritual care knowledge and skills. The current work verified that the training protocol designed in this study to enhance nurses’ spiritual health and spiritual care competencies is feasible and effective. Clinical nursing managers should use this protocol as a reference to help clinical nurses improve their spiritual health. Therefore, the results of this study have great relevance at a time when a worldwide shortage of nurses exists. The protocol developed in this study should also be used to improve nurses’ spiritual care knowledge and skills to enable them to better satisfy patients’ spiritual needs and improve their nursing quality.

### Research limitations and future research

All participants in this study included nurses in various departments of a single cancer hospital. Furthermore, to facilitate the continued provision of spiritual care training to all nurses, a considerable portion of these participants consisted of head nurses or nursing staff members in their respective departments. As a consequence, most of the nurses in this study were senior personnel with over seven years of experience. Although these individuals had a certain degree of representativeness, some uncertainty remains concerning the effectiveness of the intervention protocol when applied to nurses with less seniority. Subsequent research should therefore examine the effectiveness of the training protocol in the case of less experienced nurses. In addition, certain contents of the intervention (such as “Illness comes from the mind: Anxiety and fear will cause the functioning of qi in our bodies to shut down, thereby impeding circulation of life force, whereas joy and serenity cause the qi to function freely, enabling our life force to flow freely and create an energy field, preventing illnesses from drawing near”) is a combination of the unique elements of Chinese culture and the content of intervention with Chinese cultural characteristics. The transcultural validity of the intervention requires further consideration and verification.

Future research may focus on the following aspects: First, through the spiritual care training programme, expert instruction may boost the nurses’ perceptions of spirituality. Moreover, nurses who were originally unfamiliar with spiritual concepts became conscious that everyone has a spiritual nature and uncovered their own spiritual nature. Second, in this case, it was easier for the trainees to reveal their inner thoughts in front of trusted team members, and this process also might strengthen their ties with their colleagues and help them find positive resources and strength that they may use to cope with problems, thereby facilitating their spiritual growth while providing a platform for ongoing learning that benefited the nurses’ spiritual care competencies. Third, nurses with spiritual care experience will value the opportunity for a healthy life even more and will show greater tolerance for the challenges that they encounter. During the process of helping patients find meaning in life and overcoming adversity, nurses will also gain a greater ability to make their lives meaningful and overcome adversity, which will make this process mutually influencing and mutually reinforcing. As a consequence of the practical experience needed to assess patients’ spiritual needs in a real-world environment and provide spiritual care to patients, in addition to the positive feedback that the results of spiritual nursing brings nurses, nurses’ spiritual care competencies and personal spiritual well-being will continue to improve. Future research may evaluate these potential benefits for nurses.

## Conclusions

Nurses with spiritual health have a better ability to recognize and respond to patients’ spiritual needs and are more likely to proactively provide spiritual care to patients. Using the enhancement of nurses’ spiritual health as a point of entry, this study developed and implemented a spiritual care training protocol to improve nurses’ spiritual care. The results showed that this protocol can boost nurses’ spiritual health and spiritual care competencies. This spiritual care training protocol might be worth additional promotion and application among nurses.

## Supplementary information

**Additional file 1: Table S1**. Comparison of demographic characteristics between nurses in the two groups. **Table S2**. Nurses’ clinical spiritual care training protocol.

**Additional file 2: Table S3**. Comparison of spiritual health and spiritual care competency scores of the two groups of nurses before and after the intervention (points, $\overline{\mathrm{x}}$ ± S). **Table S4**. Comparison of spiritual health and spiritual care competency scores of the study and control group before and after the intervention (points, $\overline{\mathrm{x}}$ ± S).

## Data Availability

The data of this study can be obtained by any reasonable request. If needed, please contact the author of this article.

## Abbreviations

AIPI: Assessment, implementation, professionalization, and quality Improvement of spiritual care
ATPSC: Attitudes towards patient spirituality and communication
CFA: Confirmatory factor analysis
C-SCCS: Chinese version of the Spiritual Care Competency Scale
EFA: Exploratory factor analysis
PTS: Personal and team support
SHS: Spiritual Health Scale
