# Supplementary material for: Effectiveness of spiritual care training to enhance spiritual health and spiritual care competency among oncology nurses
Source: BMC Palliat Care. 2019 Nov 26;18:104. doi: 10.1186/s12904-019-0489-3 (PMC6880564; doi:10.1186/s12904-019-0489-3)
Supplement: Supplementary file 1 — Additional file 1: Table S1. Comparison of demographic characteristics between nurses in the two groups. Table S2. Nurses’ clinical spiritual care training protocol. [file 12904_2019_489_MOESM1_ESM.doc]

Table 1 Comparison of demographic characteristics between nurses in the two groups

| Characteristic | Study group  （n＝45） | Control group  （n＝47） | $t$or χ^2^ value | *p*-value |
| --- | --- | --- | --- | --- |
| Age（year, $\text{M}\pm SD$） | 38.07$\pm$6.52 | 36.08$\pm$7.15 | 1.39 | 0.17 |
| Working years (year, $\text{M}\pm SD$) | 18.16$\pm$7.71 | 16.27$\pm$7.94 | 1.09 | 0.28 |
| Educational level |  |  |  |  |
| Junior college or below | 0 | 2 | 5.06 | 0.08 |
| Undergraduate | 42 | 45 |  |  |
| Postgraduate or above | 3 | 0 |  |  |
| Income/month（￥） |  |  |  |  |
| ＜6000 | 0 | 6 | 8.33 | 0.04^*^ |
| 6000～ | 17 | 22 |  |  |
| 8000～ | 28 | 19 |  |  |
| Technical titles |  |  |  |  |
| Nurse Practitioner | 2 | 12 | 9.06 | 0.03^*^ |
| Supervisor nurse | 30 | 27 |  |  |
| Associate professor of nursing and above | 13 | 8 |  |  |
| Position |  |  |  |  |
| Nurse | 21 | 27 | 3.07 | 0.38 |
| Ward head nurse | 20 | 19 |  |  |
| Chief nurse and above | 4 | 1 |  |  |
| Religion |  |  |  |  |
| Yes (Christianity, 6; Buddhism,1) | 7 | 5 | 0.49 | 0.48 |
| No | 38 | 42 |  |  |
| Self-work ability evaluation |  |  |  |  |
| Excellent | 19 | 18 | 1.02 | 0.60 |
| Good | 21 | 26 |  |  |
| Qualified | 5 | 3 |  |  |

Ρ<0.05，the difference was statistically significant.

**Table 2** Nurses' clinical spiritual care training protocol

| Intervention unit and time | Intervention training topic | Content |
| --- | --- | --- |
| Unit 1 | Form a group | 1. Group pledge and sharing of feelings: Trainees should have a sense of psychological security when speaking. Participants should respect others' speech with prudence and patience. 2. Moderator's most important tasks during the group pledge are to understand the speakers' exact implied meanings and help speakers reorganize their ideas by mentioning their main points. 3. Methods for handling differences of opinion among trainees 4. Helps everyone to relax, creates a pleasant atmosphere, and ensures that all the trainees are willing to express themselves. 5. Reflective logs   (a) Goal: To help the trainees to assess their important clinical spiritual care learning experiences and achieve the instructional goals in conjunction with the trainees  (b) Reflection: Trainees submit reflective logs as directed by the supervisors and use formal methods to reflect on their learning and growth in training.  (c) Recommended content of reflective logs: Relationships with patients, family members, colleagues, household members, and other important people; expression of attitudes; factors impeding or facilitating the implementation of clinical spiritual care. et al. |
| Unit 2 | Personal spiritual growth | 1. Construction of a personal world  (a) Knowing oneself: The ideas of Freud, the hierarchy of consciousness, and theory of personality structure; the Christian belief that people are composed of a physical body, mind, responses, responsibilities, and spirit.  (b) Concept of a healthy person:  (1) A healthy person has the ability to perform everyday tasks; maintains harmonious relationships with others and appreciates others; and possesses the ability to get out of a bad mood.  (2) Five stages of mental health: normal adaptive behavior, stress-response behavior, neurotic coping behavior, abnormal psychoneurotic behavior, and psychotic behavior  (3) How to shape a person who is psychologically and behaviorally healthy. Interior methods: Self-awareness (reflection on strengths and weaknesses); self-opening (open communication with others); self-identification and acceptance; and self-respect. External methods: Leading a disciplined life; setting goals in life; handling tasks in an orderly fashion; time management; learning methods for relaxation; learning methods for preparation; being a grateful person  2. Mysteries of life  (a) True meaning of life. The reason that some people are optimistic is their ability to look at problems from the correct angle; the reason that some people are pessimistic is their restricted thinking and looking at problems from a single angle.  (b) Human beings have a collective subconsciousness. We have a natural tendency to draw close to greater and more powerful life and security and connect with spiritual power.  (c) Rules of the operation of spiritual power. Human beings possess a spirit (linking all eternities), soul (linking individuals through relationships), and body (linking human beings with the material world).  (d) Rules of interaction between the material and spiritual worlds. The internal determines the external, and we must listen to the voice of the spirit. Illness comes from the mind: Anxiety and fear will cause the functioning of qi in our bodies to shut down, thereby impeding circulation of life force, whereas joy and serenity cause the qi to function freely, enabling our life force to flow freely and create an energy field, preventing illnesses from drawing near. Fear is the murderer of human life. Human beings should retain positive energy and joyful emotions such as kindness, love, compassion, and harmony, embrace honesty and goodness, and maintain serenity, happiness, and hope, which will improve their physical and mental health.  (e) Spiritual integration. Following short-term treatment, many ill patients can obtain a complete cure by adjusting their inner power. If people cannot improve their illnesses due to emotional inhibition, them they should search for spiritual caregivers who know how to listen to help them find their potential inner power and achieve spiritual integration to counteract illness. Both health and illness can be transmitted. Heaven helps those who help themselves.  (f) Relationship between thinking and attitude. Fear is in inverse proportion to self-confidence. Aesop provided the following example: 45,000 people who encounter an epidemic die from fear. A joyful heart is good medicine. The highest state of helping others is to enable those seeking help to help themselves. The five secrets of helping others are self-knowledge, self-acceptance, self-mastery, self-liberation, and self-integration.  3. Positive life education:  (a) Positive psychology is the study of an individual character’s strengths that focuses on the establishment of a positive mood and a sense of happiness as well as emphasizes holistic development and the goal of uncovering, cultivating, and realizing individuals' strengths and potential. The positive factors promoting human development include optimism, joy, and mutual love. Actively facing life's stresses and challenges can enable us to lead fuller lives.  (b) The "happy Ferris wheel". The subject of positive psychology is well-being. Well-being includes five measurable elements: positive emotions, wholehearted commitment, interpersonal relationships, meaning, and a sense of accomplishment.  (c) Virtue and character strengths. According to the "Father of American Psychology", "If an individual can use his or her personal strengths in work, leisure, and family life, [then] that individual can easily appreciate authentic happiness and a sense of well-being". The six Chinese virtues are intelligence and knowledge, courage, spirit and transcendence, benevolence and compassion, righteousness, and moderation. |
| Unit 3 | Spiritual care cognition and practice | 1. Discussion on the concept of spiritual care 2. Spiritual care methods: birth, body, mind, society, spirit 3. Teaching spiritual care according to the content of the spiritual care cognitive scale and the spiritual care ability assessment scale 4. Spiritual Care Practice: Patients are assessed with a psychological pain thermometer when they are admitted to the hospital, and one-on-one mind care intervention is given to patients with a psychological distress greater than 4 points. On the other hand, if the patient assesses the degree of psychological distress greater than 6 points, there is a spiritual care group to conduct spiritual counseling for the patient after the discussion. |
| Unit 4 | Life-and-death education | 1. Origin of life-and-death education 2. The concepts of death of the Egyptians, Tibetans, Babylonians, Persians, Greeks, and communists 3. The relationship between death and life. Death is a continuation of life. Death helps people reflect on their lives. Death explains life. Death helps us cherish life. Death causes us to undergo an awakening. Life and death are fundamentally one entity. 4. Reasons that the Chinese fear death: fear of the unknown, fear of separation and loss, fear of the form and process of death, media reports, attachment to continuity, and unfulfilled wishes and regrets concerning the fear of being unable to make amends. 5. Methods of resolution: turning the unknown into the known; learning to accept unavoidable loss and tragedy in life; learning, accepting, and understanding human fragility and the restrictions of life; clearly understanding the meaning of eternity, and adjusting the priority of one's values on this basis; knowing gratitude for what one has and living without regret 6. Modern people's discussion of death. Influences on modern people's perception of life and death (Confucianism, Daoism, Buddhism, folk beliefs, Islam, Judaism, Christianity, and others) 7. Death education   (a) Discussion of end-of-life care and death. Case analysis: The regrettable consequences of when family members refuse to discuss death  (b) Discussing Life and Death with Equanimity (Center of Behavioral Health, University of Hong Kong). The correct preparation for death, including recollection of past details and preparing for the end of life  (c) *Unloading sorrows: A manual for helpers and self-help* (Ng and Yim). Five sentences to leave the world without regret: “I love you.” “Please forgive me.” “I forgive you.” “Thank you.” “Goodbye.”  (d) *Twenty-three Ways to Practice Saying Goodbye* (Hori). In the last moments, there are 23 practices to say goodbye, including finding the strength to live on (eight practice methods), going down the final road together in happiness (nine practice methods), and a message written from you to who will be left in the world (six practice methods). Let children participate in death education.   1. The "life" in life-and-death education. The Universal Declaration of Human Rights; nutrition and physical/mental health; health information on diet and mood; healthy and happy living equals emotional health+social health+psychological health+spiritual health 2. The "death" in life-and-death education. Discussing death with equanimity: having a good life plan |
| Unit 5 | Suicide prevention | 1. Definition and forms of suicidal behavior. Causes of suicidal behavior: Physiological and mental problems (e.g., physiological illnesses, psychological problems, and drug/alcohol abuse); social causes (family, work, school, interpersonal, social changes, and psychic contagion); and spiritual reasons (guilt and deviant beliefs). 2. Warning signs of possible suicide: withdrawal, excessively emotional reactions, aggressiveness, abuse of medication, promiscuity, abnormal diet, giving treasured items as gifts, pain, personality changes, intimidation, and fear 3. Key preventive measures: Early discovery of suicide risk factors; aggressive treatment of mental illness; maintaining contact with people with suicidal tendencies; not making discussion of suicide a taboo; asking people with suicidal tendencies whether they have suicidal ideations, suicidal impulses, or concrete suicide plans; immediately arranging referral for diagnosis by a specialist as soon as it is discovered that someone is at risk of suicide 4. Handling suicide crises: confirming that the person involved is actually committing or planning suicide; assessing the danger of the person's suicidal behavior, understanding the person's behavioral motivations; drafting an assistance plan 5. Correcting misunderstandings about suicide and understanding the true face of suicide. Most people who talk often about suicide will try to commit suicide. Signs of possible suicide: Suicidal individuals might accept guidance, although risk remains even after the immediate risk of suicide is past. Suicidal individuals include the rich and poor, men and women, and people of all ages. Suicidal individuals do not necessarily have mental illnesses or psychological problems. The suicide rate is highest among youth. The frequent mention of suicide facilitates suicide prevention. 6. How to face crises in life 7. Crises include dangers and opportunities   (b) Common elements of crises. Crises are composed of a cumulative series of events, situations of high risk, and unexpected/sudden factors.  (c) Handling methods: establishing balancing factors such as the correct understanding and analysis of the case; ensuring an adequate support network, a dependable response system, and limited handling time  (d) Characteristics of middle-aged people and crisis handling. The establishment of a sense of safety and a support network among friends; long-term psychological preparation; economic arrangements; social support |
| Unit 6 | End-of-life care | 1. The meaning of palliative end-of-life care. A team who have received special training to provide physical, mental, social, and spiritual care and support to terminal patients help patients ease their suffering and discomfort while respecting their dignity and values and enabling them to pass their remaining time in comfort and dignity 2. Promoting ethics bills concerning end-of-life care work: death education and mourning assistance; the provision of adequate consulting and referral resources 3. The ability of people engaged in end-of-life care work. Understanding of one's own feelings about death and the ability to forthrightly describe and express those feelings; the ability to understand service recipients’ ideas about death and their emotional response development stage; the ability to discern and understand society's viewpoints and discussion of death; a full knowledge of the science of death and life-and-death education; an understanding of one's own viewpoint concerning different death events and the ability to maintain a neutral position; skill at employing empathy; excellent communication skills and ability 4. Taiwan's "Five Alls" model: All subdistricts, all team members, all people, full-course, and the whole household 5. Holistic care: life, body, mind, society, spirit 6. Needs of patients receiving end-of-life care: a clear understanding of their illness; the alleviation of one's suffering; companionship; maintenance of autonomy; the maintenance of effective communication; emotional relief and support; the preservation of dignity; the recollection and integration of life experiences 7. What is a good death? No need for prolonged suffering before death; the reduction of suffering as much as possible before death; the chance to reconcile with family members and friends before death; the knowledge that one does not need to worry about family members' lives after one's death; the completion of one's family responsibilities before death; the ability to take a load off one's mind   8. End-of-life care model: past, present, future; individual, family, social relationships, and spiritual aspects |
