# Supplementary material for: Effectiveness of spiritual care training to enhance spiritual health and spiritual care competency among oncology nurses
Source: BMC Palliat Care. 2019 Nov 26;18:104. doi: 10.1186/s12904-019-0489-3 (PMC6880564; doi:10.1186/s12904-019-0489-3)
Supplement: Supplementary file 2 — Additional file 2: Table S3. Comparison of spiritual health and spiritual care competency scores of the two groups of nurses before and after the intervention (points, $\overline{\mathrm{x}}$x¯ ± S). Table S4. Comparison of spiritual health and spiritual care competency scores of the study and control group before and after the intervention (points, $\overline{\mathrm{x}}$x¯ ± S). [file 12904_2019_489_MOESM2_ESM.docx]

**Table 3** Comparison of spiritual health and spiritual care competency scores of the two groups of nurses before/after intervention (points，$\overline{\text{x}}$ $\pm$S)

| Content | |  | Before intervention | | | |  | After intervention | | | |
| --- | --- | --- | --- | --- | --- | --- | --- | --- | --- | --- | --- |
|  |  |  | Study group (n=45) | Control group(n=47) | $t$value | $\eta^{2}$ |  | Study group (n=45) | Control group (n=47) | $t$value | $\eta^{2}$ |
| Spiritual health | Total score |  | 90.29$\pm$13.12 | 91.57$\pm$12.05 | -0.49^b^ | .00 |  | 106.49$\pm$8.31 | 95.00$\pm$11.91 | 5.38^*^ | .24 |
|  | Connection to others |  | 16.51$\pm$2.19 | 17.06$\pm$2.93 | -1.02^b^ | .01 |  | 18.67$\pm$1.58 | 17.68$\pm$2.68 | 3.05^*^ | .09 |
|  | Meaning derived from living |  | 24.22$\pm$3.89 | 24.21$\pm$4.77 | 0.01^b^ | .00 |  | 27.69$\pm$2.66 | 25.43$\pm$4.44 | 2.98^*^ | .09 |
|  | Transcendence |  | 21.82$\pm$5.04 | 23.72$\pm$4.41 | 1.93^b^ | .04 |  | 27.18$\pm$3.21 | 24.57$\pm$4.58 | 3.15^*^ | .10 |
|  | Religious attachment |  | 12.62$\pm$3.82 | 10.09$\pm$4.35 | 2.97^*^ | .09 |  | 15.07$\pm$3.95 | 10.91$\pm$4.80 | 4.54^*^ | .19 |
|  | Self-understanding |  | 15.11$\pm$3.00 | 16.49$\pm$2.58 | -2.37^*^ | .06 |  | 17.89$\pm$1.90 | 16.81$\pm$3.03 | 2.06^*^ | .04 |
| Spiritual care competency | Total score |  | 79.24$\pm$20.70 | 91.81$\pm$15.68 | -3.27^*^ | .11 |  | 110.84$\pm$9.40 | 95.60$\pm$15.22 | 5.81^*^ | .27 |
|  | AIPI |  | 34.42$\pm$10.43 | 39.49$\pm$8.87 | -2.51^*^ | .07 |  | 47.73$\pm$6.12 | 41.02$\pm$7.89 | 4.57^*^ | .19 |
|  | PTS |  | 24.13$\pm$7.18 | 28.68$\pm$5.38 | -3.43^*^ | .12 |  | 36.64$\pm$3.78 | 30.91$\pm$5.41 | 5.90^*^ | .28 |
|  | ATPSC |  | 20.69$\pm$5.32 | 23.64$\pm$3.99 | -3.00^*^ | .09 |  | 26.47$\pm$2.62 | 23.66$\pm$4.02 | 3.95^*^ | .15 |

^*^ P＜0.05；^b^ P＞0.05；^a^ P=0.05; $\eta^{2}$: Eta Squared (Effect size); AIPI: assessment, implementation, professionalization and quality improvement of spiritual care; PTS: personal and team support; ATPSC: attitude toward patient spirituality and communication.

**Table 4**  Comparison of spiritual health and spiritual care competency scores of the study and control group before and after intervention (points, $\overline{\text{x}}$ $\pm$S)

| Content | |  | Study group (n=45) | | | | |  | Control group (n=47) | | | | |
| --- | --- | --- | --- | --- | --- | --- | --- | --- | --- | --- | --- | --- | --- |
|  |  |  | Before intervention | After intervention | $d$value | $t$value | $\eta^{2}$ |  | Before intervention | After intervention | $d$值 | $t$value | $\eta^{2}$ |
| Spiritual health | Total score |  | 90.29$\pm$13.12 | 106.49$\pm$8.31 | -16.20 | -8.37^*^ | 0.23 |  | 91.57$\pm$12.05 | 95.00$\pm$11.91 | -3.43 | -2.06^a^ | 0.02 |
|  | Connection to others |  | 16.51$\pm$2.19 | 18.67$\pm$1.58 | -2.16 | -8.91^*^ | 0.87 |  | 17.06$\pm$2.93 | 17.68$\pm$2.68 | -0.21 | -0.52^b^ | 0.09 |
|  | Meaning derived from living |  | 24.22$\pm$3.89 | 27.69$\pm$2.66 | -3.47 | -5.85^*^ | 0.49 |  | 24.21$\pm$4.77 | 25.43$\pm$4.44 | -1.21 | -2.02^a^ | 0.06 |
|  | Transcendence |  | 21.82$\pm$5.04 | 27.18$\pm$3.21 | -5.36 | -6.97^*^ | 0.52 |  | 23.72$\pm$4.41 | 24.57$\pm$4.58 | -0.85 | -1.20^b^ | 0.04 |
|  | Religious attachment |  | 12.62$\pm$3.82 | 15.07$\pm$3.95 | -2.44 | -5.49^*^ | 0.17 |  | 10.09$\pm$4.35 | 10.91$\pm$4.80 | -0.83 | -1.46^b^ | 0.04 |
|  | Self-understanding |  | 15.11$\pm$3.00 | 17.89$\pm$1.90 | -2.78 | -5.91^*^ | 0.77 |  | 16.49$\pm$2.58 | 16.81$\pm$3.03 | -0.32 | -0.74^b^ | 0.03 |
| Spiritual care competency | Total score |  | 79.24$\pm$20.70 | 110.84$\pm$9.40 | -31.60 | -9.74^*^ | 0.36 |  | 91.81$\pm$15.68 | 95.60$\pm$15.22 | -3.79 | -2.33^a^ | 0.01 |
|  | AIPI |  | 34.42$\pm$10.43 | 47.73$\pm$6.12 | -13.31 | -7.94^*^ | 0.36 |  | 39.49$\pm$8.87 | 41.02$\pm$7.89 | -1.53 | -1.49^b^ | 0.02 |
|  | PTS |  | 24.13$\pm$7.18 | 36.64$\pm$3.78 | -12.51 | -11.51^*^ | 0.88 |  | 28.68$\pm$5.38 | 30.91$\pm$5.41 | -2.23 | -3.22^a^ | 0.08 |
|  | ATPSC |  | 20.69$\pm$5.32 | 26.47$\pm$2.62 | -5.78 | -7.66^*^ | 0.30 |  | 23.64$\pm$3.99 | 23.66$\pm$4.02 | -0.02 | -0.04^b^ | 0.00 |

^*^ P＜0.05；^b^ P＞0.05；^a^ P=0.05; $\eta^{2}$: Eta Squared (Effect size); AIPI: assessment, implementation, professionalization and quality improvement of spiritual care; PTS: personal and team support; ATPSC: attitude toward patient spirituality and communication.
